# Building mud castles: a perspective from brick-laying termites

**DOI:** 10.1038/s41598-017-04295-3

**Published:** 2017-07-05

**Authors:** Nikita Zachariah, Aritra Das, Tejas G. Murthy, Renee M. Borges

**Affiliations:** 1 Centre for Ecological Sciences, Indian Institute of Science, Bangalore, 560012 India; 2 Centre for Neuroscience, Indian Institute of Science, Bangalore, 560012 India; 3 Department of Civil Engineering, Indian Institute of Science, Bangalore, 560012 India

## Abstract

Animal constructions such as termite mounds have received scrutiny by architects, structural engineers, soil scientists and behavioural ecologists but their basic building blocks remain uncharacterized and the criteria used for material selection unexplored. By conducting controlled experiments on *Odontotermes obesus* termites, we characterize the building blocks of termite mounds and determine the key elements defining material choice and usage by these accomplished engineers. Using biocement and a self-organized process, termites fabricate, transport and assemble spherical unitary structures called boluses that have a bimodal size distribution, achieving an optimal packing solution for mound construction. Granular, hydrophilic, osmotically inactive, non-hygroscopic materials with surface roughness, rigidity and containing organic matter are the easiest to handle and are crucial determinants of mass transfer during mound construction. We suggest that these properties, along with optimal moisture availability, are important predictors of the global geographic distribution of termites.

## Introduction

Animals build structures using exogenous or endogenous materials^1^. These structures with their varied functions such as thermoregulation or protection from predation are important targets of natural selection since their structural and functional efficacy determine the reproductive success of their builders^2^. While aspects of animal constructions such as architecture^3^, regulation of nest internal environment^4–6^ and collective construction behaviour^7^ have been studied, less is known about the basic building blocks or bricks of construction and how material properties affect their choice and usage.

In social organisms, construction involves many individuals working in tandem with often clear division of labour^8^. In the soil environment, such organisms build nests usually by burrowing, e.g. excavation of pelleted particulates by ants^9^ or by assemblage of excavated soil by biocementation possibly coordinated by chemical signals^3, 10, 11^, e.g. termite mounds^12^. During burrowing, stability of pellets is required until they are transported out of the excavated nest^9^. However, in construction, longer term agglomeration of particulates is required to ensure the strength and stability of the overall structure; to increase strength, soil pellets are, therefore, often cemented using body secretions^13^. Termite mounds are excellent examples of collective construction and biocementation. They are dominant features of several landscapes, three orders of magnitude greater in height than individual termite dimensions (see Supplementary Fig. S1) and can endure for time spans ranging from several decades (RM Borges, pers. observ.) to centuries^14^. To achieve high strength, termites mix soil with their saliva and possibly excretions^13^ resulting in a 10-fold increase in the soil particle agglomerate strength^15^. Termites agglomerate the finer fraction of soil particles into unitary structures (bricks) called boluses, which they employ for mound construction^15^. Previous studies on termites have examined different soil types in mound and soil sheeting construction^16–18^. Construction in a termite mound can be envisioned as a three-part process: material selection, transport and binding or assemblage. We present data that have bearing on all three construction stages and, using our results taken together, we define the crucial determinants of mound construction by termites.

**Figure 1:**
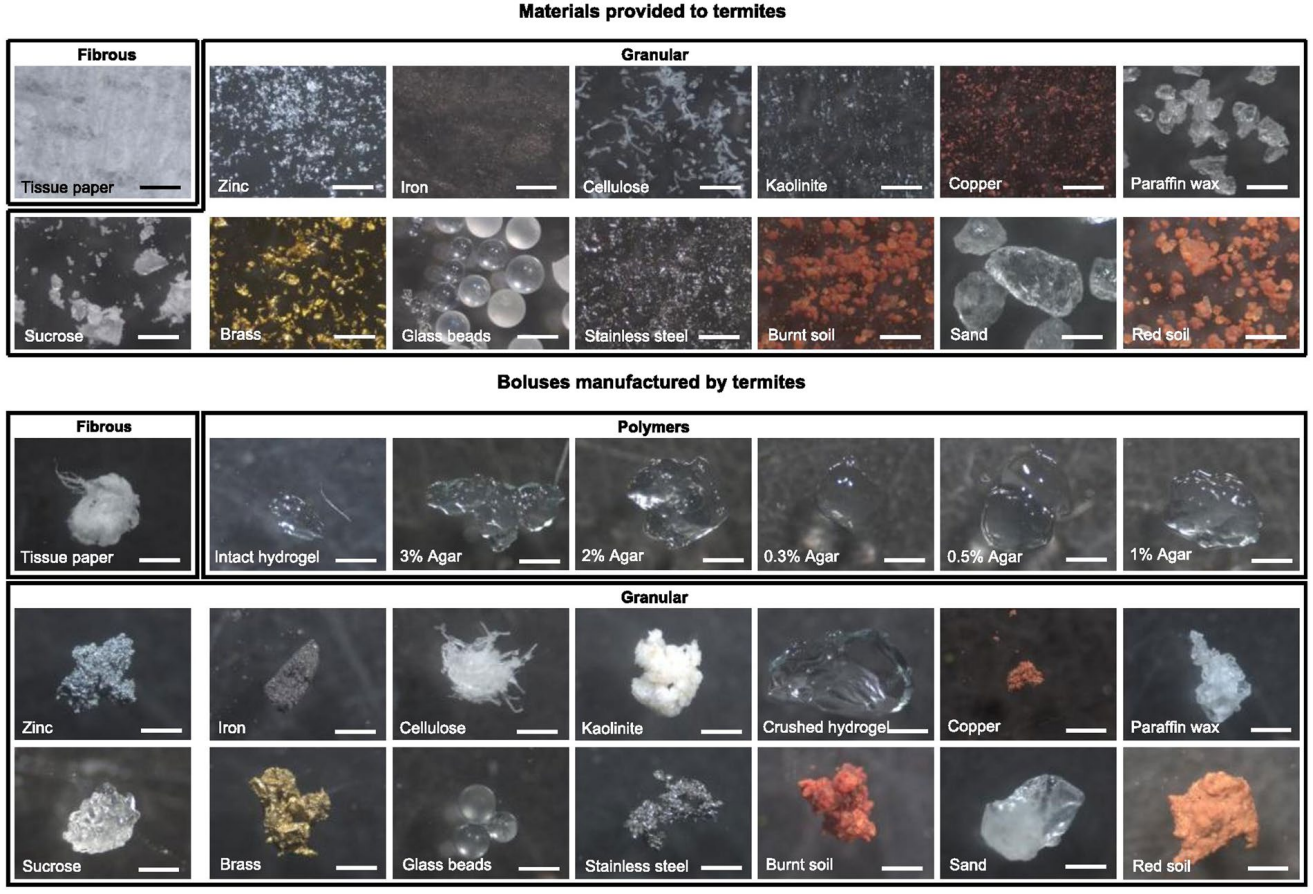
Boluses made by major workers with different materials (material indicated on each image). No boluses were made with 0.1%, 0.2% agar and sodium chloride. Scale bars represent 0.5 mm.

Our study termite species is *Odontotermes obesus* and the study site was the Indian Institute of Science Campus in Bangalore, India (see Methods). We found that only major and minor worker castes of termites (not soldiers) agglomerated the locally available red soil into boluses, which are densely compacted structures with a range of soil particle sizes (Fig. 1; see Supplementary Figs S2 and S3a; Supplementary Video S1). These boluses are the basic building blocks of construction and are analogous to bricks used in human construction.

## Results

### Effect of particle size and termite caste on bolus volume

**Figure 2:**
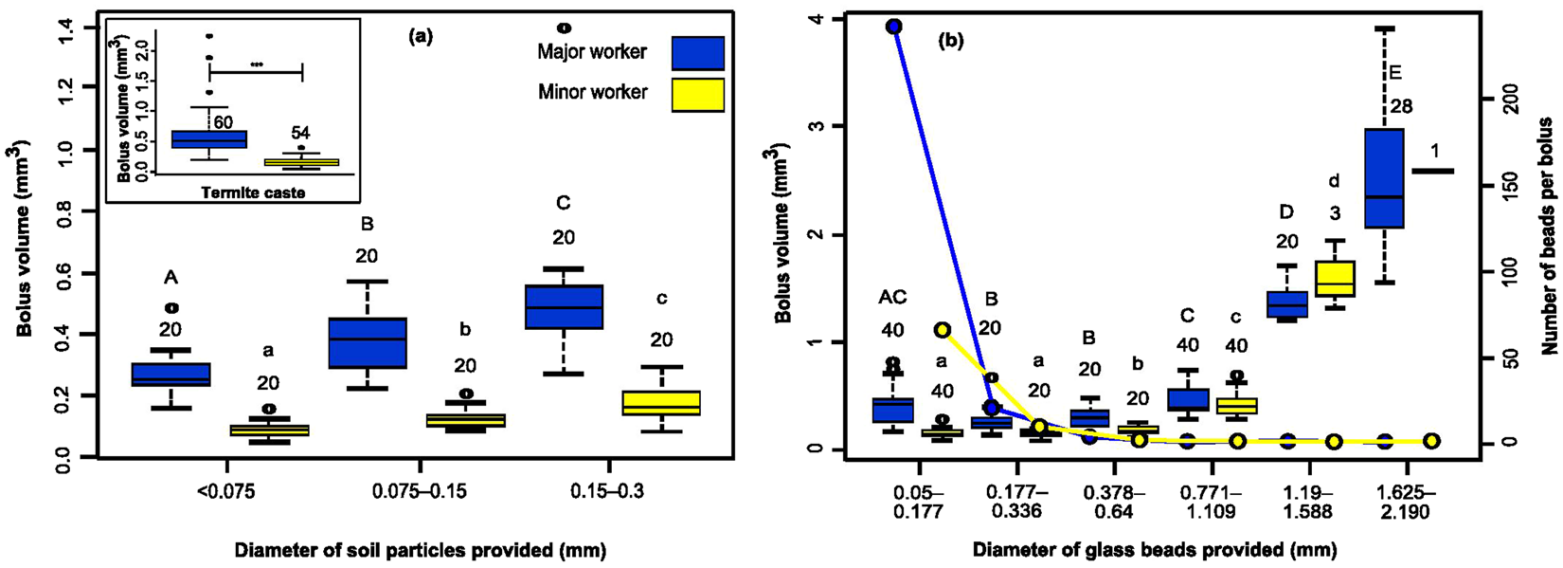
Boluses made with different materials. (**a**) Boluses made with soil of different particle sizes; inset shows boluses made *in situ* at 5 termite mounds; (**b**) Boluses made with glass beads of different sizes; dots represent number of beads per bolus; box plots with horizontal lines indicating median, bottom and top of the box indicating 25th and 75th percentiles respectively, and whiskers indicating either the maximum value or 1.5 times the inter-quartile range, whichever is smaller. Numbers above box plots indicate sample sizes. Capital and small alphabets indicate within-caste significant differences obtained from Tukey’s HSD tests for major and minor workers respectively.

There was a bimodal distribution of bolus sizes between castes when we collected boluses from both major and minor worker castes during repairs of experimentally induced breaches in the external walls of active mounds. The volume of boluses fabricated by major workers was 3.7 times larger than those of minor workers (Fig. 2a inset; see Supplementary Table S1; data from all mounds pooled together). GLMM analysis with log_e_(Bolus Volume) ~ Caste + (1|Mound/Caste) (see Methods for details) showed very low inter-class correlation for Mound (ICC = 0.039) and Caste:Mound (ICC = 0.056) suggesting negligible contribution of these variables in explaining the total variance (see Supplementary Table S2), indicating that caste was the major determinant of differences in bolus volume between major and minor workers. Soil boluses were 0.25–0.3 times the head volume for both castes (Fig. 2a inset; see Supplementary Fig. S2; Supplementary Table S1), and weighed 17% and 9% of the body weights of major and minor workers respectively (see Supplementary Table S1).

When termites were experimentally offered red soil and glass beads of varied sizes in the presence of water (see Methods, Supplementary Table S3; Supplementary Video S2), bolus size increased with increase in particle size provided, but for any given particle size, boluses made by major workers were always larger than those of minor workers (Fig. 2a,b; see Supplementary Table S1). For soil and glass bead boluses made in the lab, an analysis of variance was performed with log_e_(Bolus Volume) ~ Caste + Particle Diameter. There was a significant effect of caste and particle size on bolus volume for both soil and glass bead boluses (Soil boluses: Caste: F_1,116_ = 448.74, p ≪ 0.001; Particle Diameter: F_2,116_ = 48.09, p ≪ 0.001; Glass bead boluses: F_1,285_ = 484.0, p ≪ 0.001; Particle Diameter F_5,285_ = 193.7, p ≪ 0.001). We therefore examined the effect of increasing particle size within a caste. We found that with increasing soil particle size/glass bead size, there was an increase in bolus volume in the examined size range (Fig. 2a,b; Tukey HSD post-hoc tests for each caste separately for soil and glass bead boluses; see Supplementary Tables S4, S5, S6 and S7). For each caste, bolus size increased with increase in glass bead diameter provided till termites stopped carrying boluses (Fig. 2b). The increase in glass bead diameter was accompanied by a decrease in the number of beads per bolus till termites carried only one bead as a single bolus. The weights and volumes of boluses of the largest soil particle size tested were close to those of boluses made with natural red soil *in situ* (see Supplementary Table S1) indicating that the entire range of particle sizes normally available to termites *in situ* was adequately explored experimentally. Deposited termite secretions (and/or excretions) act as biocement between the glass beads (see Supplementary Fig. S2), which also must bind particles in the soil boluses.

### Effect of material properties on termite mound construction

**Figure 3:**
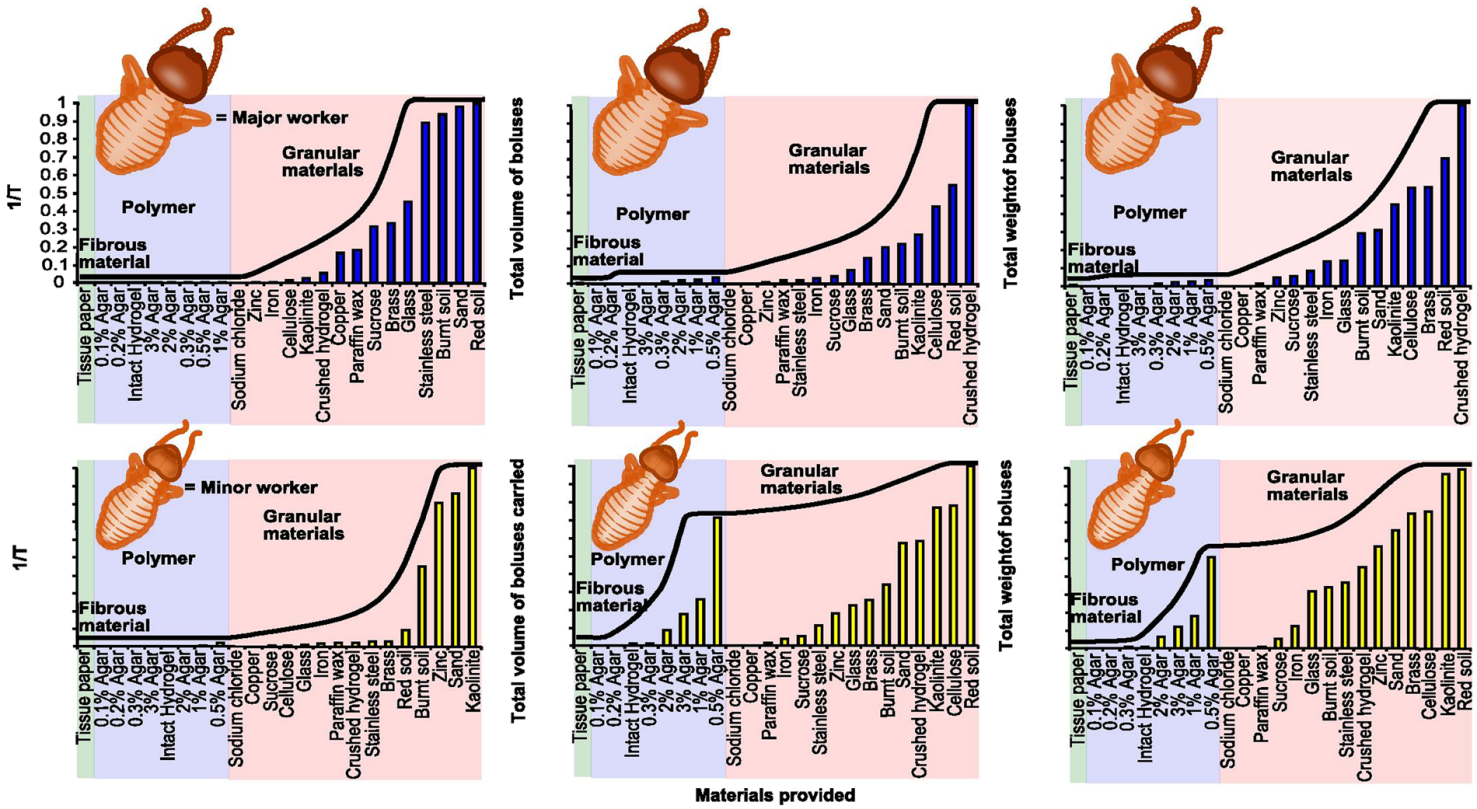
Ease of handling for different materials. Ease-of-handling parameters normalized for the maximum value in each category.

In an attempt to understand the effect of material properties on the process of mound construction, major and minor termite worker castes were offered a wide range of materials in the presence of extraneous moisture, and their latency to bolus making (T) and the rate of bolus making were measured for each offered material. From this, the reciprocal of latency (1/T), and the total volume and weight of material carried were calculated as measures of the ‘ease of handling’ of materials (see Methods). These parameters have bearing on the economics of mass transfer during mound construction. We found that materials were handled differently based on their properties. Granular materials were aggregated into boluses while pieces were excavated from polymer lawns (agar and intact hydrogels) or cut from fibrous materials (Fig. 1, see Supplementary Video S3). The transition from fibrous materials and polymers to granular materials was accompanied by a steep rise in ease of handling, indicating that granular materials were transported fastest and to the greatest extent (Fig. 3). Minor workers transported polymers such as agar which major workers largely ignored possibly because minor workers might be more efficient in walking on agar surfaces or excavating boluses from them. Within granular materials, red soil, sand, minerals (kaolinite), and crushed hydrogel were some of the most readily handled materials. Metal powders and glass beads had intermediate latency in handling and rate of transport. When offered steel balls in the permissible range of size and weight, termites did not utilize them for making boluses until the surface roughness of the beads was increased (see Methods), suggesting that frictional forces are also important in handling ease. Hydrophobic (copper, paraffin wax), osmotically active and hygroscopic (sodium chloride) materials had the lowest ease of handling (even lower than that for most polymers), were used by very few individuals, and the boluses made were very small in size (Fig. 1). Some materials were very easy to handle (e.g. red soil, burnt soil (see Methods for description of burnt soil), sand, glass beads, kaolinite, crushed hydrogel), some were difficult (e.g. intact hydrogel, 3% agar, tissue paper, copper) and some were not handled at all (NaCl, 0.1% and 0.2% agar) (Fig. 3). Neither caste constructed boluses with agar concentrations below 0.3% (viscosity = 9.1 Pa.s, tensile strength = 7.6 Pa), and both were unable to walk on 0.1% and 0.2% agar owing to the low rigidity of these substrates (see Supplementary Fig. S4; Supplementary Table S8). Mortality was observed in less than 90 minutes when NaCl was offered as building material.

Latency to start making boluses was similar for red and burnt soil but the rate of making boluses from red soil was close to double that for burnt soil (Fig. 3). Boluses made with burnt soil were much more fragile than boluses made up of unburnt soil (unpublished results) resulting in low transport of burnt soil boluses when compared to red soil. The rate of making boluses by major workers was highest for crushed hydrogel (measured in terms of volume and weight of boluses carried), probably because termites carried one piece of crushed hydrogel as one bolus and therefore did not have to spend time in excavating and aggregating particles with their saliva. Thus, for all practical purposes, crushed hydrogel can be treated as a granular material.

**Figure 4:**
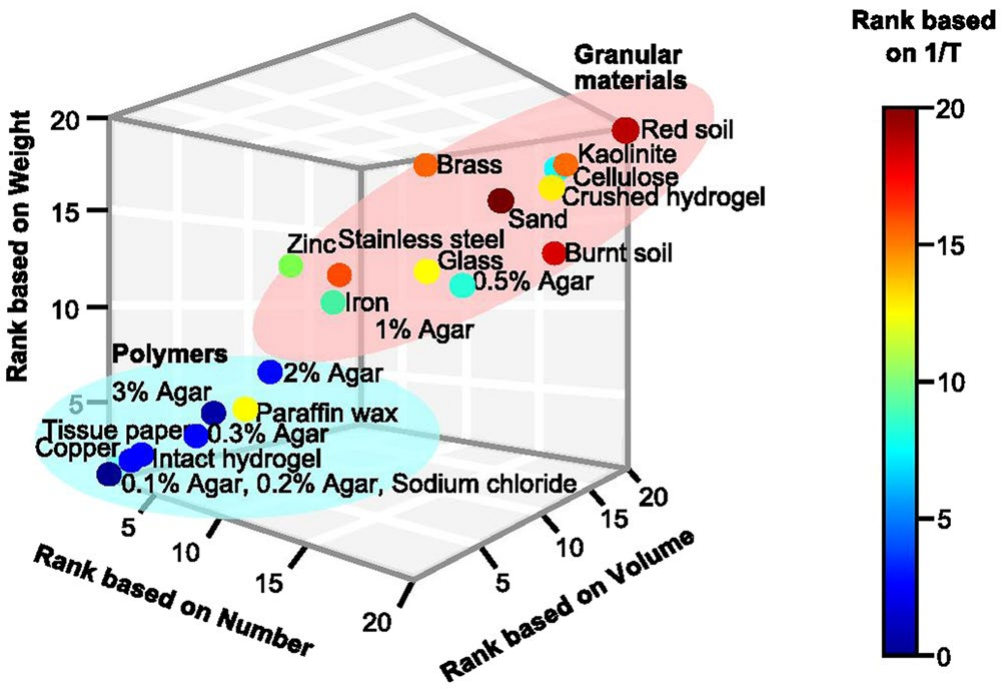
Combined ranking of materials by individuals of both castes based on four criteria. 1/T represents 1/Time taken to start making boluses; Number represents total number of boluses made in 20 minutes; Volume represents total volume of boluses made in 20 minutes, and Weight represents total weight of boluses carried in 20 minutes.

Granular, hydrophilic and osmotically inactive materials received the highest ranks based on four criteria (latency in fabricating the first bolus, total number, volume and weight of boluses transported and assembled within a fixed time) (Fig. 4). High concordance was observed between these criteria for both termite castes (unweighted ranks: major workers: W = 0.9, χ² = 83.2, p ≪ 0.01; minor workers: W = 0.9, χ² = 84.2, p ≪ 0.01) and weighted ranks (Cohen’s weighted kappa; see Supplementary Table S9). In a simultaneous choice between red soil and glass beads as a test of differential handling of a familiar versus unfamiliar building material (see Supplementary Fig. S5), termites used both materials equally efficiently and showed no preference towards red soil which is a familiar material (Wilcoxon signed rank test for major workers: V = 11, p = 0.1055; for minor workers: V = 30, p = 0.8457), suggesting that granularity overrides familiarity in terms of material choice. Thus, termites fabricate boluses as a constitutive activity and this behaviour appears to be hard-wired in them, such that they will agglomerate and transport materials of all types, preferentially granular, in the presence of optimal moisture content to aid the cementation process.

### Achieving optimal packing of boluses

**Figure 5:**
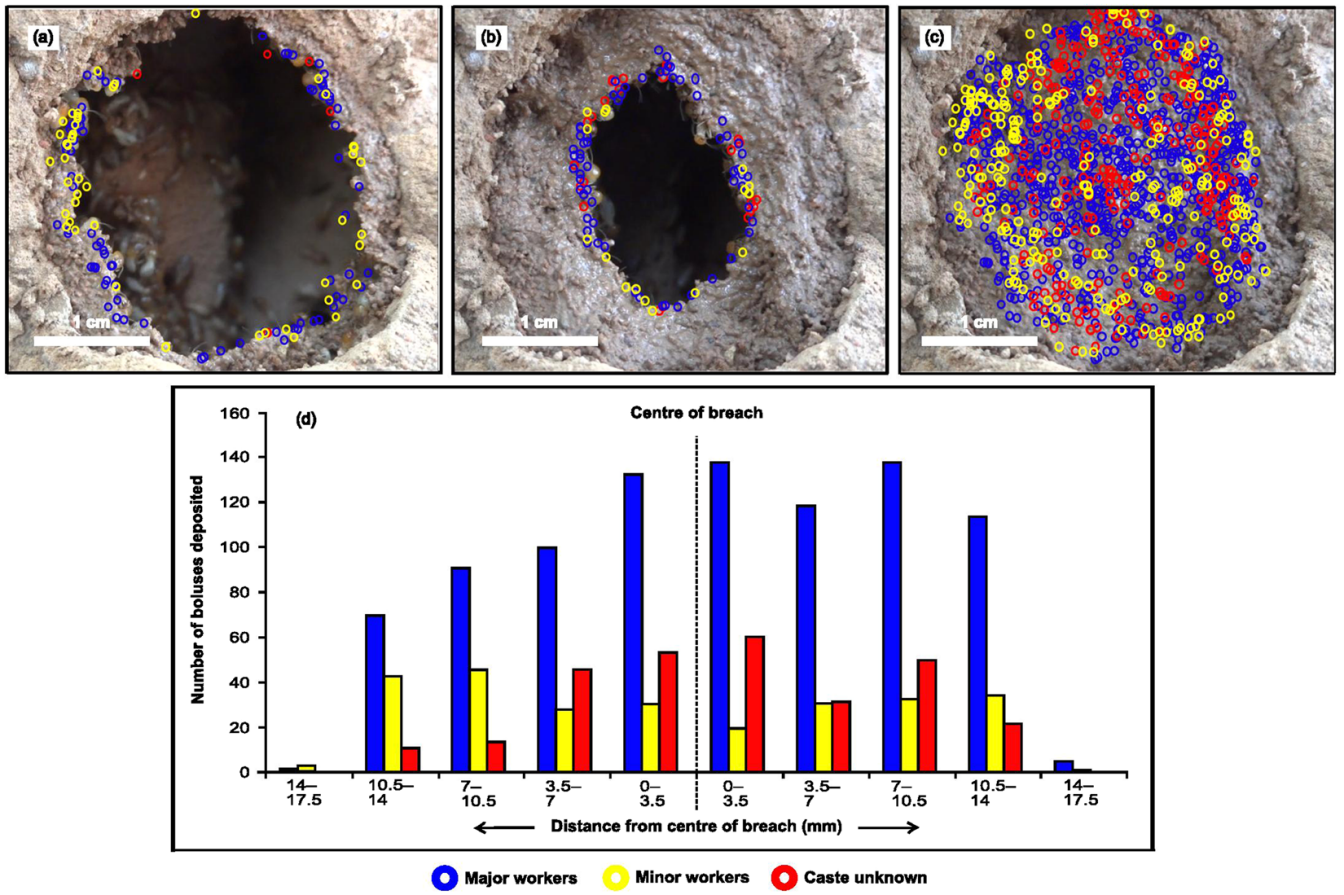
Placement of boluses by major and minor workers during breach repair in a termite mound as recorded by videography from a fixed location. (**a**) Boluses deposited during 1^st^ minute of breach repair; (**b**) boluses deposited during 8^th^ minute of breach repair; (**c**) boluses deposited during the entire process of breach repair; some circles indicating bolus depositions are partially overlapping and can obscure others from view; (**d**) boluses deposited during the entire process collapsed on to the horizontal axis in ten equal bins from the centre of the breach. When the worker caste was not clearly visible, it is indicated as “caste unknown”. Breach repair occurs in a circular manner and boluses made by major and minor workers were interspersed, leading to high packing efficiency.

Since there was a significant difference in the size of red soil boluses carried by major and minor workers (Fig. 2), which is a likely consequence of the size difference between castes (see Supplementary Table S1), it is possible that minor workers assemble their small boluses in the voids formed between the large boluses of major workers and thus achieve high material packing efficiency during mound construction. In order to test this, we made an intentional breach in a mound and the process of repair was video recorded from a fixed location (see Supplementary Video S4). The video was split into frames and on each frame the boluses deposited by major and minor workers were marked. The breach was repaired in a circular manner with boluses being deposited along the entire circumference of the breach (Fig. 5). More importantly, boluses made by minor workers were interspersed with those of major workers in accordance with an optimal packing solution during construction (Fig. 5c,d).

## Discussion

In this study we characterized the basic building block of termite mound and studied the behaviour associated with material usage for construction. *In situ* and *ex situ* experiments with soil showed that bolus size depended both on termite caste and soil particle size. We found that sizes of soil boluses made in the lab were comparable to the ones made *in situ* (Fig. 2a and inset). At the mound, termites face two different conditions — construction of a mound extension or breach repair is carried out in the presence of light but remodelling of the interior regions of the mound takes place in the dark. We found that in spite of the difference in lighting conditions, boluses made *in situ* were comparable in size to the ones made *ex situ* (Fig. 2a and inset). Also, as evident from Fig. 2, boluses made with soil and glass beads offered in the same size range (<0.75–0.3 mm diameter for soil and 0.05–0.336 mm diameter for glass beads) were comparable in size (below 1 mm³). Therefore bolus making appears to be hard wired in termites; the size of bolus made is independent of the location (*in situ* bolus making versus manipulative experiments in lab), the purpose for which it is made (breach repair versus protection from desiccation by tunnelling in moist soil in petri dishes), and the shape of particle provided (irregular soil particles versus spherical glass beads).

Granular, hydrophilic, osmotically inactive, non-hygroscopic materials with surface roughness, rigidity and containing organic matter were the easiest to handle and define the scope of mound construction by termite brick-layers that also includes the economics of mass transfer. The large spread in the ease of handling of granular materials based on all four criteria (Fig. 3) may be due to the differences in the size, shape, chemical composition, rigidity and presence of organic matter in different kinds of granular materials making some more favourable than others. Boluses devoid of organic matter had a lower fabrication rate (comparison between red soil and burnt soil boluses) probably because organic matter, like salivary secretions, also contributes to cementation. In an earlier study^15^ (and unpublished results), we found that workers of *O. obesus* can use red soil in a wide range of moisture contents (15–60% moisture per unit dry weight of soil) which spans the entire range of moisture contents from the plastic limit to the liquid limit of the red soil (17% and 33% moisture per unit dry weight of soil respectively). Below 15% moisture content, termites were not able to aggregate soil into boluses with their saliva and above 60% they were unable to walk on the surface, corroborating our results obtained with other materials in this study that termites use materials in the presence of moisture as long as they are able to locomote on the surface. These results taken together indicate that granularity and availability of water appear to be the most important criteria for facilitating material usage.

Granular materials can be packed in a wide range of densities based on grain size distribution and grain shape^19, 20^, and can also be cemented together into contact-bound or matrix structures^21^ which form the basis of ventilation and thermoregulation in termite mounds^4, 6^. Such materials offer the choice of achieving a range of packing efficiencies within and across boluses. We found that the boluses made by major and minor workers that have a bimodal size distribution were deposited in a closely interspersed manner during mound breach repair. It is well known that such a distribution of particle sizes ensures an optimal random close packed structure^19^, which ensures greater efficiency at the ensemble level. Moreover, this does not require a fixed ratio of larger and small particles (major and minor worker boluses in this case) and works reasonably with varying ratios. Previous work has also shown a ten-fold increase in strength of termite mound soil due to biocementation^15^. Therefore, biocementation coupled with a packing of boluses, and matrix suction due to partially saturated conditions^15^ with a bimodal size distribution guarantees the required amount of strength and durability.

Finally, collective construction has long been studied to gain an understanding of how rules at lower levels can produce an adaptive emergent outcome. Collective animal construction is decentralized and often relies greatly on stigmergy, where individuals, without interacting directly, coordinate their activities by modification and sensing of a shared environment. Examples include possible use of coordinating pheromones in nest building in termites^10^ and in ants^3, 11^. Attempts have been made to understand such mechanisms in termite mound construction using robotics, where an individual robot perceives the position of preformed, solid “bricks” and of other robots in the vicinity, and then decides where to place the next brick^7^. The “bricks” used in these studies consisted of a single type of prefabricated building material. Our work provides the first empirical evidence that in a termite mound there are two distinct types of bricks (boluses) and that the behaviour associated with the making of these bricks and their rate of transfer depends on the material system provided. We also propose a mechanism of stigmergy where boluses deposited by one caste (say major workers) form a scaffold and are perceived by the other caste; the other caste deposits its boluses in the voids between these boluses based on positional information. This tandem construction behaviour with boluses of two size classes deposited by major and minor workers is one way in which termites can efficiently solve the optimal packing problem and must contribute to the phenomenal strength and stability exhibited by the termite mound. Attempts have also been made to extend robotic construction to unstructured terrains using amorphous construction materials such as foam that react by changing shape to conform to their environment. Amorphous materials circumvent alignment and attachment restriction faced by pre-fabricated building materials and also compensate for uncertainty and measurement errors^22^. During termite mound construction, boluses are deposited in the presence of moisture which allows them to coalesce and form an amorphous but reasonably uniform mass (see Supplementary Fig. S3).

Since this study was conducted only on one species of higher termite, i.e. *Odontotermes obesus*, caution must be exerted in extending these results beyond higher termites. Nevertheless, we believe that the material properties explored in this paper can play important roles in mound and gallery building by a wide range of termite species. Thus, while the absolute values for ease of handling parameters shown in Fig. 3 may change based on the species and its feeding and nesting habits^23^, the general principles of material choice and utilisation are likely to remain the same.

We conclude that the behaviour of making boluses is innate to the worker castes of termites, with clear caste-based differences and a strong preference for granular materials. Granular, hydrophilic and osmotically inactive nature/non-hygroscopic materials, particle size, weight, surface roughness, rigidity and presence of organic matter are important determinants of the material systems termites can employ for making boluses. Taken together, these variables are crucial predictors for mound construction (Fig. 4, region highlighted in red). These parameters, apart from favourable climatic conditions and availability of water, are necessary and sufficient for terrestrial mound construction by termites. The soil stratum of any geographic region which satisfies these conditions is likely conducive for mound construction by termites. All else being equal, places where termite mounds are unexpected include marshes (where the soil forms a slurry with water and termites will be unable to walk on such surfaces), deserts (sand grains might be too large to be handled, and there is low organic matter and moisture preventing adhesion of soil particles into boluses) and salt deserts/pans (due to presence of osmotically active and hygroscopic materials). Our findings can therefore also help predict the geographical distribution of mound-building termites at local and regional scales, a matter of considerable interest, especially since termites are known to modulate soil–water ecosystem processes and thereby climatic effects^24^.

## Methods

### Study species and site

*Odontotermes obesus* is a fungus-farming, mound-building termite widely distributed in the Indian subcontinent^25^, and its castle-shaped, buttressed mounds are up to several metres in height (see Supplementary Fig. S1). The study site (Indian Institute of Science Campus in Bangalore, India) has a residual red soil (referred to as red soil in this paper) formed from the physical and chemical weathering of gneissic bedrock and is classified as inorganic clay of low plasticity^26^. It contains kaolinite as the dominant clay mineral followed by montmorillonite; its non-clay mineral fraction is comprised of quartz, mica and feldspar. The sand, silt and clay-sized fractions in this soil are 43%, 34% and 23% respectively^26^. Boluses made by *O. obesus* with this soil are nearly spherical in shape but with irregular surfaces (see Supplementary Video S1). We conducted a series of preliminary investigations to shortlist the parameters that need to be tested in order to develop an understanding of bolus (“brick”) making. Since termites carry boluses using their mouth parts, and morphologically different termite castes (major and minor workers) have dissimilar head sizes, we expected that termite caste and soil particle size would each have an effect on bolus size. Termites also encounter materials of varying rigidities in nature and are exposed to a wide range of materials such as granular materials (soil and sand), polymers (root exudates of plants) and fibrous materials (dead plant leaves). Therefore, we tested the effect of material rigidity on bolus size and the ease of handling of different material classes. To study the effects of increasing particle size and increasing material rigidity on bolus construction, we offered a range of glass beads and agar concentrations to termites, instead of soil, to avoid the effects of differences in clay minerals and organic matter content of different soil fractions.

### Effect of termite caste on bolus size

We made intentional breaches in five termite mounds and boluses were collected directly from the mouth parts of major and minor workers on needles, dried and stored separately. 10–20 boluses were collected from each caste at all five mounds, brought to the lab, photographed (Axiocam MRC) and measured using ImageJ software. X-ray tomography was also conducted on individual boluses in the Advanced Facility for Microscopy and Microanalysis at the Indian Institute of Science using a ZEISS Xradia 520 Versa 3D X-ray microscope with 4x objective and air as the filter. We calculated the weights of boluses using the density of soil–water mixture as 1.44 mg/mm³ (this corresponds to the *in situ* moisture content of boluses which is about 17% per unit dry weight of soil or 14.5% per unit wet weight of soil^15^).

### Effect of particle size on bolus size

One termite mound was chosen arbitrarily for these experiments. Termites collected during the process of breach repair at the mound were brought to the lab and kept in petri dishes on moist tissue paper to prevent mortality from desiccation. All experiments were completed within 12 hours of termite collection. Both major and minor worker castes of termites were provided moist red soil (26% moisture per unit wet weight) of three different particle sizes (less than 0.075 mm, 0.075–0.15 mm and 0.15–0.3 mm, n = 20 petri dishes; one termite per dish) in mini-petri dishes (35 mm diameter) and were allowed to make boluses. The moisture content provided was above the plastic limit of red soil and also slightly above the moisture content at which termites were found to deposit soil at the mound^15^ and was chosen because termites made boluses faster at this moisture content. Experiments were conducted in the dark as termites are averse to light^27^. Termites made boluses which they deposited on the walls of the petri dishes. One bolus was collected per petri dish, dried and measured as mentioned before. Termites were also provided spherical glass beads in sizes ranging from 0.05–2 mm (Hindustan Glass Beads, ethanol washed and dried) (see Supplementary Table S3). Glass beads are a standard surrogate for soil and have been widely employed in studies of granular materials^28^. Boluses were directly collected from termite mouths since glass bead boluses, once deposited on top of each other or on the walls of petri dishes, become individually indistinguishable. Therefore, all experiments with glass beads were performed under normal light. Boluses were measured as before. Body weights and head volumes of major and minor workers were also measured in both experiments.

### Effect of material rigidity on bolus fabrication

Both termite castes were offered agar (0.1%–3%) in petri dishes and were allowed to make boluses in the dark. We could not cast agar gels above 3% concentration. The dynamic viscosity of these agar concentrations was determined by dynamic flow rheometry with a cone and plate geometry. Viscosity of agar was plotted against shear strain rate for each agar percentage (see Supplementary Fig. S4).

### Effect of material properties on their ‘ease of handling’

In order to understand the predictors of material usage in all phases of bioengineering by termites — material choice, transport and assembly — major and minor termite workers were offered a range of construction materials that included fibrous materials, polymers and granular materials (Fig. 1) in the presence of extraneous moisture (Supplementary Fig. S6). The amount of moisture required for all stages of construction depended on the wettability of the material and was experimentally optimised (Supplementary Fig. S6). We employed two measures to evaluate material choice and transport which provide estimates of ease of handling with bearing on the economics of material transfer. We used 1) latency (T) and its reciprocal (1/T) to determine how soon termites initiated bolus-making for a particular material (low 1/T values indicate very high latency) and 2) the rate of bolus-making (total number, volume and weight carried within a fixed observation period of 90 min in a 35 mm petri dish) as a measure of transport. Bolus-making rate, therefore, is an estimate of the amount of time and energy spent in carrying a given amount of material and reflects the economics of mass transfer for a given material system. In case termites did not start making boluses in 90 minutes, latency (T) was scored as infinity and its reciprocal (1/T) was scored as zero. Besides red soil, burnt soil (red soil from which all organic matter has been combusted by igniting the soil at 440 °C in a muffle furnace^29^) was also offered to examine the effect of soil organic matter on handling ease. Hydrogel beads were offered to termites in intact and crushed forms (referred to as crushed hydrogel). Hydrophilic materials were moistened with water and offered to termites since termites cannot make boluses with dry materials (Supplementary Fig. S6). For hydrophobic materials (Supplementary Video S3), moist tissue was placed in one corner of the petri dish to ensure humidity. For tests with paraffin wax, the material was grated and crushed in liquid nitrogen to very fine powder. One representative bolus for each material and each termite caste was measured to determine its volume (Fig. 1) from which total volume and weight carried in 20 minutes were determined for all materials. Low ease of material handling and fragility of boluses without organic matter precluded measurement of a large number of boluses for volume calculations. Only agar boluses, due to their stability, allowed measurement of 9–20 boluses for volume calculations. Steel balls in the permissible range of size and weight were provided to termites which they did not use; the same beads were rubbed against sand paper to increase roughness, cleaned with ethanol and provided to termites.

### Effect of familiarity on material choice

In our previous experiments, all granular materials were handled easily by termites. Of these materials, some are likely familiar to termites in evolutionary time (e.g. soil, sand, kaolinite) and some are clearly unfamiliar (e.g. glass beads, metal powders, crushed hydrogel). To determine if there is an inherent preference in termites for a familiar granular material in construction, we performed a choice experiment between a familiar material (red soil) and an unfamiliar material (glass beads). Termites were provided red soil (800 mg soil mixed with 200 mg water; see Supplementary Fig. S6) and glass beads (800 mg) in a 35 mm petri dish. Tissue paper (35 mm × 9 mm; 130 mg) was placed between the two materials to prevent them from mixing and 652﻿ mg water was added to moisten the tissue (78% moisture per unit wet weight) and glass beads (19% moisture) (see Supplementary Fig. S6). This was done to ensure that glass beads and tissue paper were saturated with water. (Tissue paper absorbs water above 75% (see Supplementary Fig. S6) and withdrew water from adjacent glass beads leaving them unsaturated, thereby making them unfavourable for termites.) One termite was released in the middle of the petri dish and observed for 90 minutes (n = 10 each for major and minor workers). The total number of boluses made with soil and glass beads was counted.

### Statistical analysis

We analysed the data using the software package R v.3.3.3. Data were tested for normality using the Shapiro-Wilk test. All bolus volumes were log_e_ transformed to achieve normality of residuals. For boluses made *in situ* (Fig. 2 inset), GLMM was employed^30^ (using the lmer function in the lmerTest package) with log_e_(Bolus Volume) as response variable, Caste as fixed effect and Mound ID as random effect. Caste was also nested within Mound ID. For boluses made by major and minor workers *ex situ* with soil and with glass beads of different diameters (Fig. 2a,b), all termites were collected from a single mound; therefore, mound ID was not considered as a predictor. In the absence of any random variable an analysis of variance was performed with log_e_(Bolus Volume) ~ Caste + Particle Diameter for both soil and glass bead boluses. Tukey’s HSD post-hoc tests were performed for each caste separately for soil and glass bead boluses. Concordance between various parameters indicating ease of handling boluses was calculated using Kendall’s coefficient of concordance (W) and Cohen’s weighted kappa (with squared weights using the kappa2 function in the irr package).

### Data availability

The data that support the findings of this study are available in the Supplementary Information and also from the corresponding author upon request.

## Electronic supplementary material

Supplementary Video S1

Supplementary Video S2

Supplementary Video S3

Supplementary Video S4

Supplementary Information
