# Supplementary material for: Building mud castles: a perspective from brick-laying termites
Source: Sci Rep. 2017 Jul 5;7:4692. doi: 10.1038/s41598-017-04295-3 (PMC5498601; doi:10.1038/s41598-017-04295-3)
Supplement: Supplementary file 5 — Supplementary Information [file 41598_2017_4295_MOESM5_ESM.pdf]

Building mud castles: a perspective from brick-laying termites

Nikita Zachariah[1], Aritra Das[2], Tejas G. Murthy[3], Renee M. Borges[1, *]

[1]Centre for Ecological Sciences, Indian Institute of Science, Bangalore 560012, India

[2]Centre for Neuroscience, Indian Institute of Science, Bangalore 560012, India

[3]Department of Civil Engineering, Indian Institute of Science, Bangalore 560012, India

* Corresponding author; Email: renee@ces.iisc.ernet.in

Supplementary Figures:

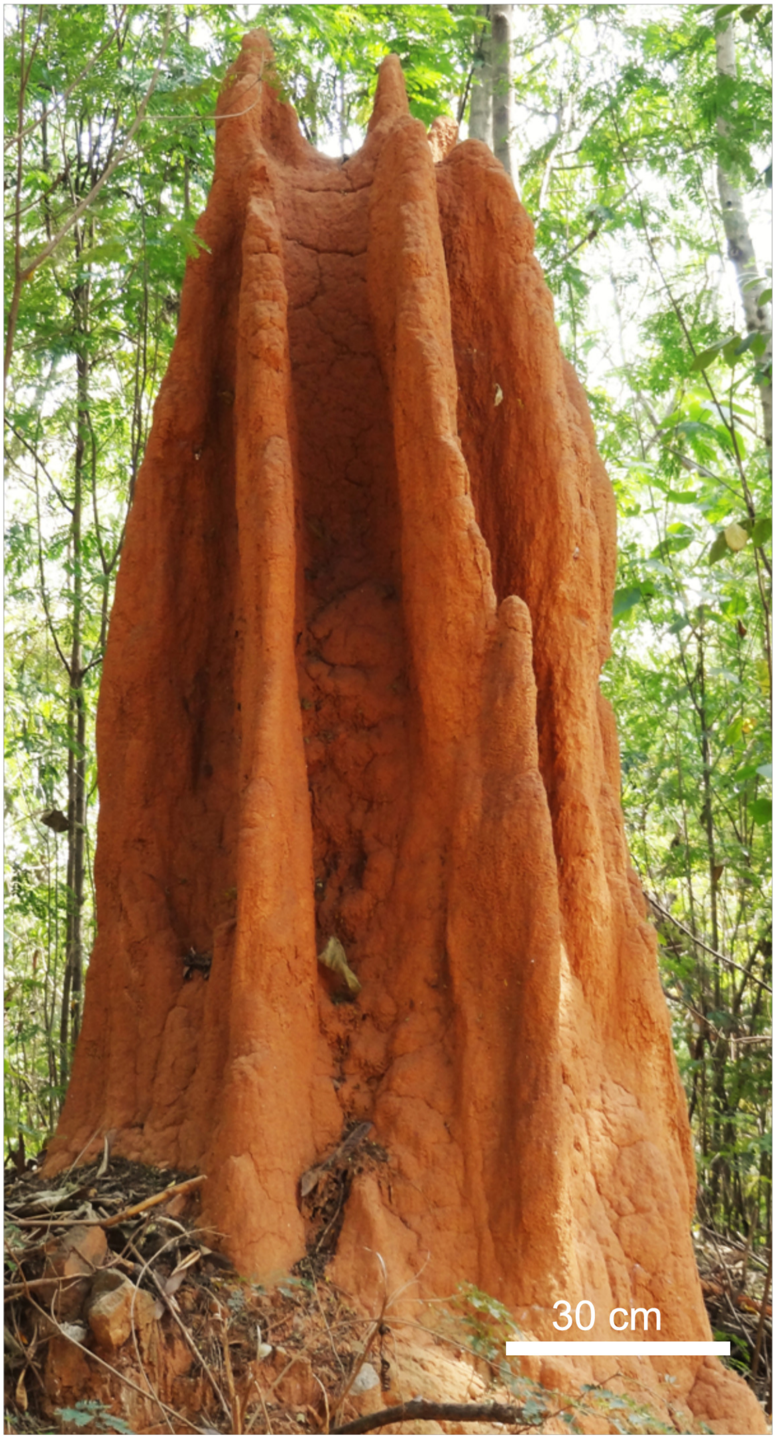

Figure S1. Mound of *Odontotermes obesus*.

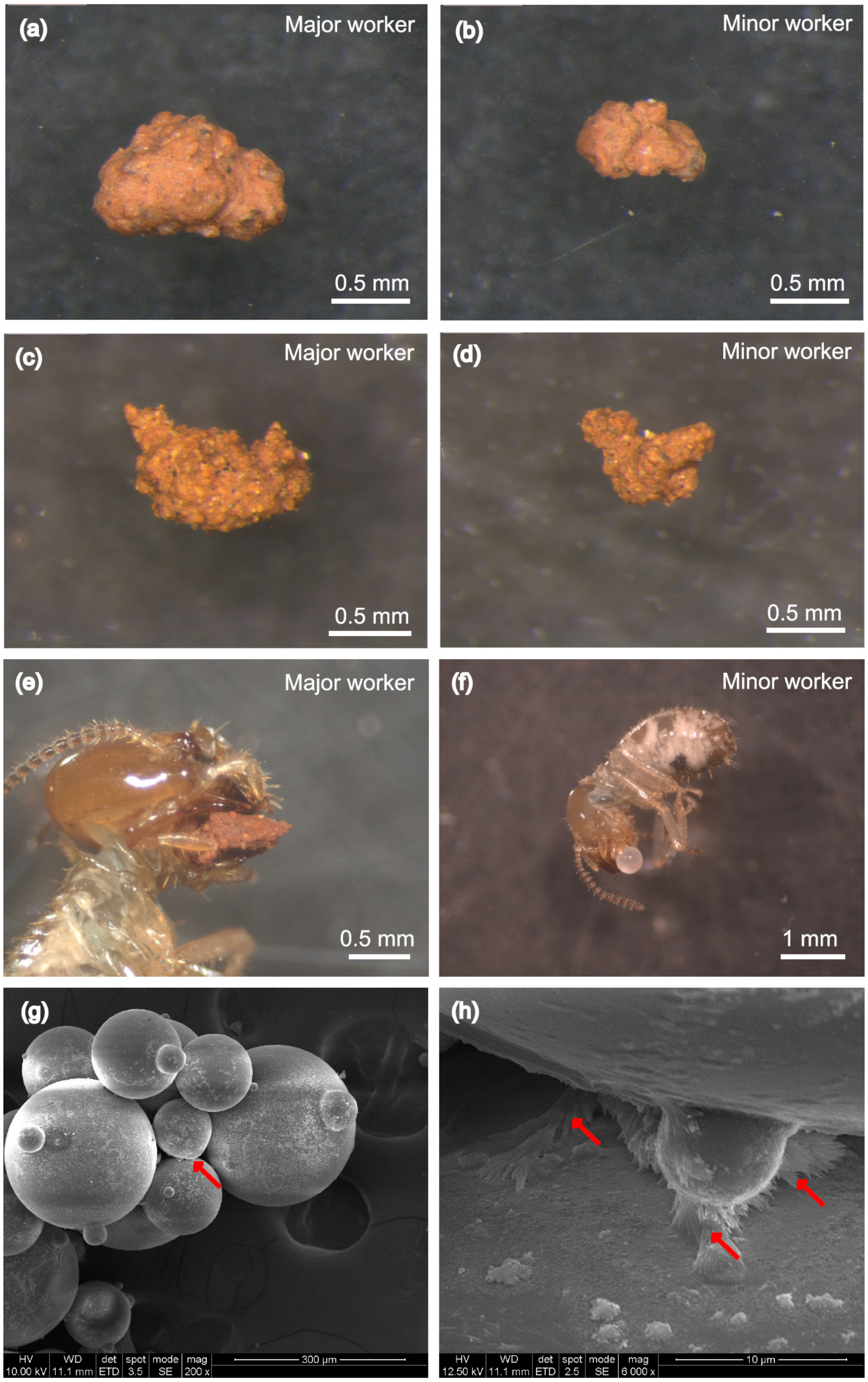

Figure S2. Boluses made with soil and glass beads. (a–b) Boluses collected from the mound; (c–d) Boluses made in the lab with < 75μ soil particle size; (e) Major worker snap frozen with soil bolus in its mouth; (f) Minor worker snap frozen with glass bead bolus in its mouth; (g–h) Electron micrographs of glass bead bolus. Arrows indicate salivary depositions between beads (g–h).

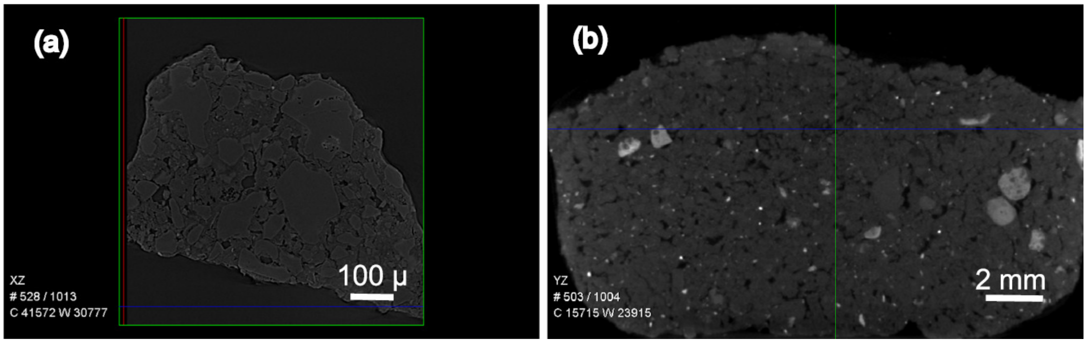

Figure S3. Section through microtomograph of (a) major worker bolus (Video S1) showing packing of soil particles within a bolus and (b) piece of termite mound wall showing packing of boluses within a mound. Boluses merge together during mound construction and become individually indistinguishable.

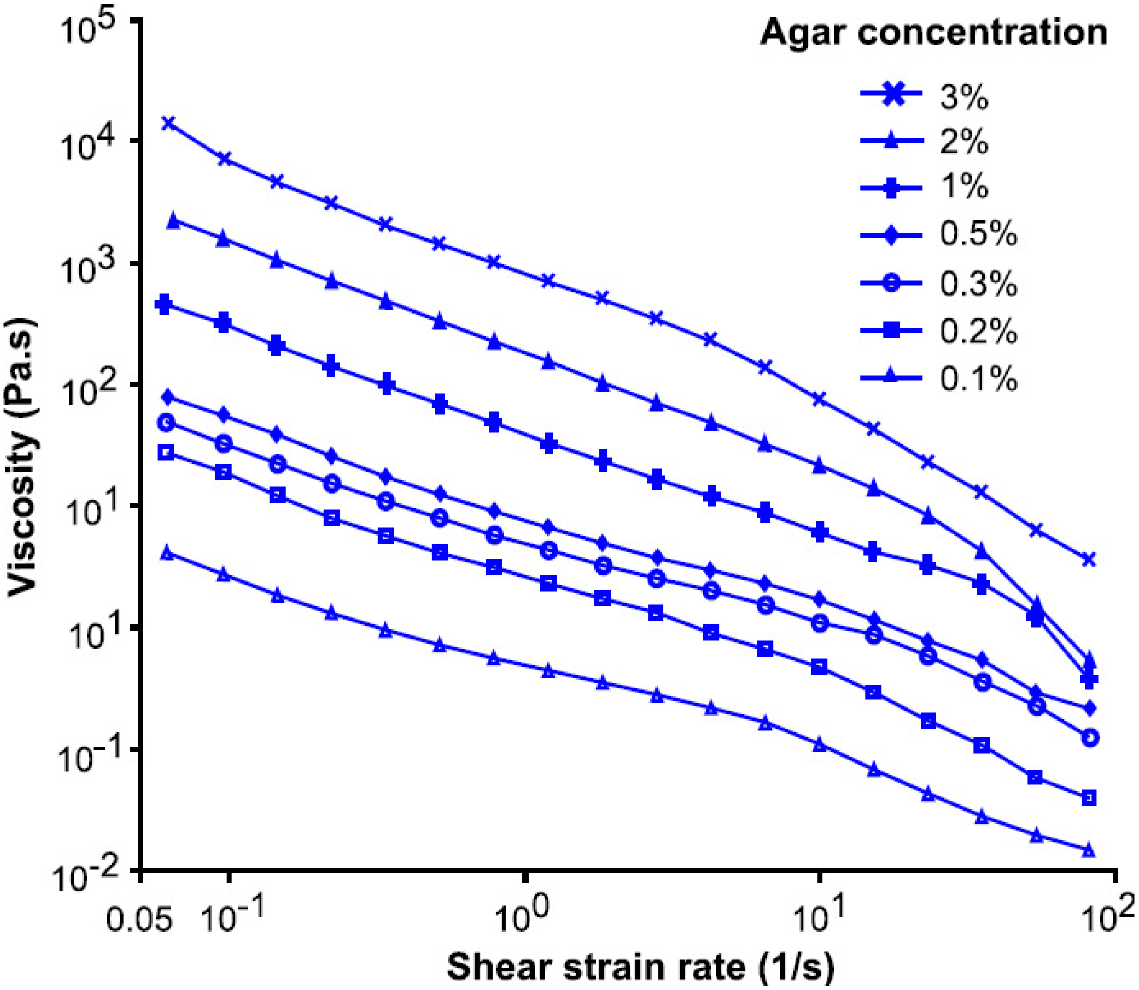

Figure S4. Change in viscosity of agar with respect to shear strain rate.

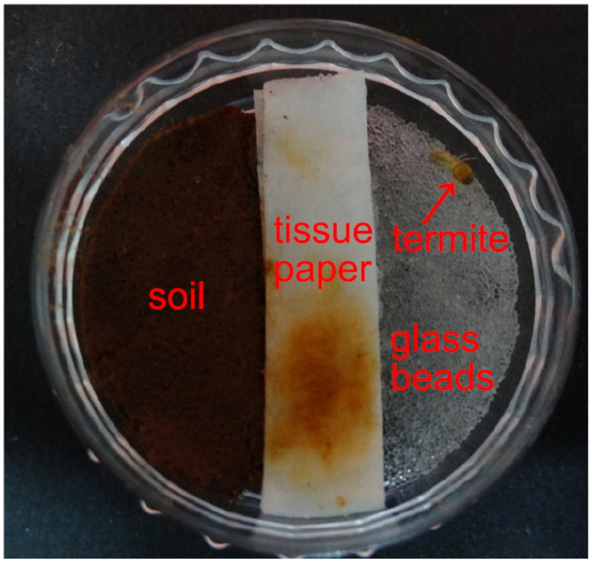

Figure S5. Behavioural arena used for studying choice between soil and glass beads.

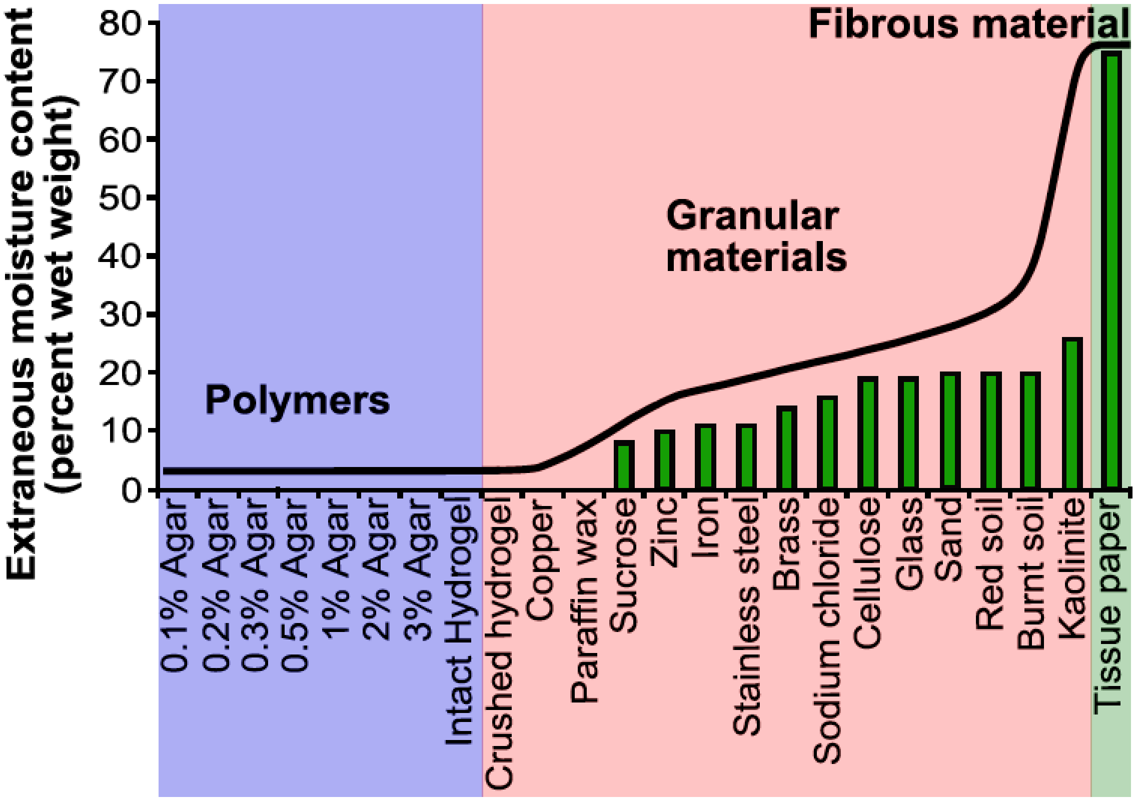

Figure S6. Extraneous moisture added to different materials while offering them to termites in order to study the ease of handling.

Supplementary Tables:

Table S1. Boluses made with soil *in situ* and *ex situ* (a) and glass beads (b). For soil boluses mean ± s.d. have been indicated. For glass bead boluses only the largest boluses carried have been indicated.

a. Soil boluses (all values mean ± s.d.)

Site	Particle size	Within-caste comparisons										Across-caste comparisons
		Major worker					Minor worker					Bolus volume or weight for major worker/ Bolus volume or weight for minor worker
		Volume of bolus			Weight of bolus		Volume of bolus			Weight of bolus		
		Absolute volume (mm³)	Bolus volume/ Head volume	Increment in size from previous treatment	Absolute weight (mg)	Bolus weight/ Body weight	Absolute volume (mm³)	Bolus volume/ Head volume	Increment in size from previous treatment	Absolute weight (mg)	Bolus weight/ Body weight	
in situ	Surrounding soil	0.59 ± 0.36	0.25	n.a.	0.85 ± 0.52	0.17	0.16 ± 0.07	0.31	n.a.	0.23 ± 0.09	0.09	3.65
ex situ	< 75 µ	0.27 ± 0.07	0.11	n.a.	0.37 ± 0.10	0.08	0.09 ± 0.03	0.18	n.a.	0.13 ± 0.03	0.05	2.95
	75–150 µ	0.38 ± 0.10	0.16	1.40 times	0.52 ± 0.14	0.10	0.13 ± 0.03	0.24	1.40 times	0.17 ± 0.05	0.07	2.97
	150–300 µ	0.52 ± 0.23	0.22	1.35 times	0.70 ± 0.31	0.14	0.17 ± 0.05	0.33	1.34 times	0.23 ± 0.07	0.09	2.99

b. Glass bead boluses

Within-caste comparisons										Across-caste comparison
Major worker					Minor worker					
Max. bead diameter carried (mm)	Max. volume carried (mm³)	Max. bolus volume/ mean head volume	Max. weight carried (mg)	Max. bolus weight/ Mean body weight	Max. bead diameter carried (mm)	Max. volume carried (mm³)	Max. bolus volume/ mean head volume	Max. weight carried (mg)	Max. bolus weight/ Mean body weight	Max. volume or weight for major worker/ Max. volume or weight for minor worker
2.116	4.96	2.09	12.89	2.60	1.753	2.82	5.35	7.33	2.86	1.76

Volume of head: Major worker = 2.37 ± 0.17 mm³; Minor worker = 0.53 ± 0.05 mm³; n=20

Body weight: Major worker = 4.96 ± 0.36 mg; Minor worker= 2.57 ± 0.37 mg; n=20

Table S2. Results of Gaussian GLMM applied to *in-situ* bolus data (Random effect variance: Caste:Mound = 10 groups, Variance 0.012, Std. Dev. 0.11; Mound = 5, Variance 0.009, Std. Dev. 0.096)

Variable	Beta Estimate	Std. Error	t value	Pr(>|t|)
(Intercept)	-0.661	0.0865	-7.639	<<0.001
Caste Minor Worker	-1.249	0.1086	-11.502	<<0.001

Table S3. Volume of bead size and bead weight for different ranges of glass beads provided.

Bead diameter (mm)	Bead volume (mm^3)	Bead weight (in mg)	Percent water added to beads (wet weight)
0.05–0.177	$6.5*10^{-5}$–$2.9*10^{-3}$	$1.6*10^{-4}$–$7.3*10^{-3}$	20%
0.177–0.336	$2.9*10^{-3}$–$2.0*10^{-2}$	$7.3*10^{-3}$–$5.0*10^{-2}$	20%
0.378–0.64	$2.8*10^{-2}$–0.1	$7.1*10^{-2}$–0.3	19%
0.771–1.109	0.2–0.7	0.6–1.8	19%
1.19–1.588	0.9–2.1	2.2–5.2	17%
1.625–2.190	2.2–5.5	5.6–13.7	15%

Table S4. Tukey's HSD post-hoc test for soil boluses made *ex-situ* by major workers ($\alpha = 0.05$).

**Comparison**	**Mean Diff.**	**95% CI of diff.**	**Summary**
Particle Diameter: 0.075 – 0.15 mm vs. <0.075 mm	0.332	0.11 to 0.55	<0.01
0.15 – 0.3 mm vs. <0.075 mm	0.608	0.39 to 0.83	<<0.001
0.15 – 0.3 mm vs. 0.075 – 0.15 mm	0.276	0.06 to 0.49	<0.01

Table S5. Tukey's HSD post-hoc test for soil boluses made *ex-situ* by minor workers (α = 0.05).

Comparison	Mean Diff.	95% CI of diff.	Summary
Particle Diameter: 0.075 – 0.15 mm vs. <0.075 mm	0.338	0.12 to 0.55	<<0.001
0.15 – 0.3 mm vs. <0.075 mm	0.620	0.41 to 0.83	<<0.001
0.15 – 0.3 mm vs. 0.075 – 0.15 mm	0.282	0.07 to 0.49	<0.01

Table S6. Tukey's HSD post-hoc test for glass bead boluses made by major workers (α = 0.05).

Comparison	Mean Diff.	95% CI of diff.	Summary
0.177 – 0.336 vs. 0.05 – 0.177	-0.470	-0.76 to -0.18	<<0.001
0.378 – 0.64 vs. 0.05 – 0.177	-0.368	-0.65 to -0.08	<0.01
0.771 – 1.109 vs. 0.05 – 0.177	0.188	-0.04 to 0.42	>0.05
1.19 – 1.588 vs. 0.05 – 0.177	1.417	1.13 to 1.70	<<0.001
1.625 – 2.190 vs. 0.05 – 0.177	2.007	1.75 to 2.26	<<0.001
0.378 – 0.64 vs. 0.177 – 0.336	0.101	-0.23 to 0.43	>0.05
0.771 – 1.109 vs. 0.177 – 0.336	0.657	0.37 to 0.94	<<0.001
1.19 – 1.588 vs. 0.177 – 0.336	1.887	1.56 to 2.22	<<0.001
1.625 – 2.190 vs. 0.177 – 0.336	2.477	2.17 to 2.78	<<0.001
0.771 – 1.109 vs. 0.378 – 0.64	0.556	0.27 to 0.84	<<0.001
1.19 – 1.588 vs. 0.378 – 0.64	1.785	1.46 to 2.11	<<0.001
1.625 – 2.190 vs. 0.378 – 0.64	2.375	2.07 to 2.68	<<0.001
1.19 – 1.588 vs. 0.771 – 1.109	1.229	0.94 to 1.51	<<0.001
1.625 – 2.190 vs. 0.771 – 1.109	1.819	1.56 to 2.08	<<0.001
1.625 – 2.190 vs. 1.19 – 1.588	0.59	0.29 to 0.89	<<0.001

Table S7. Tukey's HSD post-hoc test for glass bead boluses made by minor workers (α = 0.05).

Comparison	Mean Diff.	95% CI of diff.	Summary
0.177 – 0.336 vs. 0.05 – 0.177	0.060	-0.22 to 0.34	0.99
0.378 – 0.64 vs. 0.05 – 0.177	0.393	0.111 to 0.67	0.0012
0.771 – 1.109 vs. 0.05 – 0.177	1.395	1.17 to 1.62	<<0.001
1.19 – 1.588 vs. 0.05 – 0.177	2.857	2.24 to 3.47	<<0.001
1.625 – 2.190 vs. 0.05 – 0.177	3.368	2.33 to 4.41	<<0.001
0.378 – 0.64 vs. 0.177 – 0.336	0.333	0.01 to 0.66	0.04
0.771 – 1.109 vs. 0.177 – 0.336	1.334	1.05 to 1.62	<<0.001
1.19 – 1.588 vs. 0.177 – 0.336	2.797	2.16 to 3.43	<<0.001
1.625 – 2.190 vs. 0.177 – 0.336	3.308	2.55 to 4.36	<<0.001
0.771 – 1.109 vs. 0.378 – 0.64	1.001	0.72 to 1.28	<<0.001
1.19 – 1.588 vs. 0.378 – 0.64	2.464	1.83 to 3.10	<<0.001
1.625 – 2.190 vs. 0.378 – 0.64	2.975	1.92 to 4.03	<<0.001
1.19 – 1.588 vs. 0.771 – 1.109	1.462	0.85 to 2.07	<<0.001
1.625 – 2.190 vs. 0.771 – 1.109	1.973	0.93 to 3.01	<<0.001
1.625 – 2.190 vs. 1.19 – 1.588	0.512	-0.67 to 1.70	>0.05

Table S8. Viscosity and tensile strength of agar.

Sr. No.	Agar percentage	Viscosity (Pa.s)	Tensile strength (Pa)
**1**	0.1	0.8	0.7
**2**	0.2	4.9	3.1
**3**	0.3	9.1	7.6
**4**	0.5	14.7	11.2
**5**	1	81.0	47.1
**6**	2	399.3	168.0
**7**	3	1995.2	715.8

Table S9. Cohen's weighted kappa (squared weights) for concordance between parameters determining ease of handling. (1/T: reciprocal of latency; Number: Total number of boluses carried in 20 minutes on start of bolus making; Volume: Total volume of boluses carried in 20 minutes on start of bolus making; Weight: Total weight of boluses carried in 20 minutes on start of bolus making). N = 24 for each test.

Caste (worker)	Criteria for Comparison	Kappa	z	p-value
Major	1/T, Number	0.28	2.56	< 0.05
Major	1/T, Volume	0.32	2.86	< 0.01
Major	1/T, Weight	0.31	2.78	< 0.01
Major	Number, Volume	0.56	2.95	< 0.01
Major	Number, Weight	0.58	2.88	< 0.01
Major	Volume, Weight	0.85	4.24	<< 0.01
Minor	1/T, Number	0.18	2.03	< 0.05
Minor	1/T, Volume	0.27	2.56	< 0.05
Minor	1/T, Weight	0.3	2.98	< 0.01
Minor	Number, Volume	0.43	2.49	< 0.05
Minor	Number, Weight	0.38	1.9	> 0.05
Minor	Volume, Weight	0.88	4.55	<< 0.01

Supplementary Video Legends:

Video S1. Microtomograph of a bolus made by a major worker, mounted on a needle. The tomograph shows the 3-dimensional features of the bolus**.** White pointed material in the tomograph is a needle on which the bolus was mounted.

Video S2. Termites carrying boluses with the help of mouthparts and first pair of legs.

Video S3. Bolus making with different classes of materials. (a) granular and hydrophilic (red soil); (b) polymer (agar); (c) fibrous (tissue paper); (d) granular and hydrophobic (copper).

Video S4. Process of breach repair by termites. After recruitment of termites at the site, the closure of breach took 16 minutes. Diameter of breach was 3 cm.
